# Utilizing bio-synthesis of nanomaterials as biological agents for controlling soil-borne diseases in pepper plants: root-knot nematodes and root rot fungus

**DOI:** 10.1186/s12870-024-04760-y

**Published:** 2024-02-15

**Authors:** Rehab Y. Ghareeb, Elsayed B. Belal, Nagwa M. M. El-Khateeb, Basma A. Shreef

**Affiliations:** 1 Plant Protection and Biomolecular Diagnosis Department, Arid Lands Cultivation Research Institute (ALCRI), City of Scientific Research and Technological Applications (SARTA, City), Alexandria, Egypt; 2 Agricultural Microbiology, Agricultural Botany Department, Faculty of Agriculture, Kafrelsheikh University, 33516, Kafr El-Sheikh, Egypt

**Keywords:** ZnO-NPs, Green synthesis, Fusarium, Nematode, SEM, EDX, TEM, Gene expression

## Abstract

The utilization of *Trichoderma longibrachiatum* filtrate as a safe biocontrol method for producing zinc nanoparticles is a promising approach for managing pests and diseases in agricultural crops. The identification of *Trichoderma* sp. was achieved through PCR amplification and sequencing of 18s as ON203115, while the synthesis of ZnO-NPs was accomplished by employing Trichoderma filtration. The presence of ZnO-NPs was confirmed by observing a color change to dark green, along with the use of visible and UV spectrophotometers, and the formation and chemical structure of ZnO-NPs were examined. Direct exposure to ZnO-NPs exhibited a significant inhibitory effect on the growth of *Fusarium oxysporum* at 80.73% compared with control. Also, the percent mortality of *Meloidogyne incognita* second juveniles stage (J2s) results showed 11.82%, 37.63%, 40.86%, and 89.65% after 6, 12, 24, and 72 h, respectively in vitro. Disease resistance was assessed in the greenhouse against *M. incognita* and *F. oxysporum* using the drench application of ZnO-NPs. The application of ZnO-NPs significantly reduced the disease severity of *F. oxysporum* and improved the quality and quantity of sweet pepper yield. In addition, the application of ZnO-NPs to *M. incognita* resulted in a significant reduction in the number of nematode galls, egg masses per root, eggs/egg mass, and females by 98%, 99%, 99.9%, and 95.5% respectively.

Furthermore, it was observed that the application of ZnO-NPs to pepper plants not only inhibited the growth of *F. oxysporum* and *M. incognita*, but also promoted the recovery of pepper plants as indicated by improvements in stem length by 106%, root length 102%, fresh weight 112%, root fresh weight 107%, and leaf area 118% compared to healthy control plants. Additionally, real-time PCR application and DD-PCR technique revealed that the application of ZnO-NPs stimulated the secretion of certain enzymes. These findings suggest that the biosynthesized ZnO-NPs possess anti-nematode and antifungal properties, making them effective for protecting plants against *M. incognita* and *F. oxysporum* invasion in soil. This study significantly contributes to our understanding of the nematicidal and fungicidal activities of ZnO-NPs in suppressing soil-borne diseases.

## Introduction

The sweet pepper (*Capsicum annuum* L.), a member of the vegetable family Solanaceae, is widely grown in Egypt and throughout the world. Egypt has a total of 41,047 ha under cultivation, producing 623,221 tonnes annually 1.2. Sweet peppers have great nutritional value of in terms of vitamins, antioxidants, and other compounds may contribute to their economic significance around the world. Therefore, one of the goals in agriculture in many nations is to improve sweet pepper crop production [1–3]**.**

Root rot is a serious soil-borne disease in Pepper (*Capsicum annum* L.) caused mainly by *Fusarium* sp. [4]. According to, [5, 6] the subfamily of Hymenophora is the main cause of pepper root rot disease. Members of this family include: *F. oxysporum Schlecht, F. equiseti (Corda) Sacc., F. solani, F. verticillioide, F. vasinfectum Atk.* and *F. moniliform***.** The genus Meloidogyne has a wide geographical distribution and is commonly found to infect pepper crops. Rising crop losses have been attributed to its spread, which is primarily brought on by the planting of sweet peppers in new agricultural areas [7].

The use of chemical controls against plant pathogenic diseases is often effective. However, improper use of fungicides can lead to environmental pollution and increased herbicide resistance in plant pathogens [8]. To overcome these adverse effects, several studies have concluded that biological controls produced by microorganisms are considered less hazardous for controlling plant pathogens compared to traditional chemical treatment [9].

*Trichoderma* spp. is an important bioagent; almost 20 species of the genus *Trichoderma* act against many soil-borne diseases as well as foliar plant pathogens. *T. harzianum, T. koningii, T. viride, T. atroviride, T. pseudokoningii, T. longibrachiatum, T. hamatum, T. polysporum* and* T. reesei* are the most important species, which act as potential antagonists [10].

The primary mechanisms through which Trichoderma species act include antibiosis, competition for resources and space, mycoparasitism, and the induction of defense mechanisms. Antibiosis refers to the production and release of both secondary and primary metabolites that hinder the growth and development of pathogens. In terms of metabolite production and release, around 390 non-volatile metabolites have been discovered in *Trichoderma* spp., but only a small number have been identified in *T. virens*, such as gliotoxin, gliovirin, heptelidic acid, and viridin. Sepedonin, cathequin, caffeic acid, ferulic acid, 3, 4, 15-scirpenetriol and naematolin [11].

According to [12] nanotechnology is used to refer to technology at the nanoscale which is defined as materials that are 1–100 nanometres in size. These materials are known as nonmaterial and have been found to be useful in suppressing plant diseases (biocontrol) and improving plant growth by reducing nutrient loss. Bacteria, algae, plants, diatoms and fungi are considered NP factories for their properties as reducing agents and stabilizers [13]. Fungi have special interest due to their rapid mycelia growth offering an increased surface area, easy handling of the biomass and production on a large scale. In addition, their ability to secrete significant amounts of proteins would amplify the nanoparticle synthesis productivity [14].

The scientific community has recently become interested in studying ZnO nanostructures because of their biocompatibility. They hope to learn more about their cytotoxicity, interactions with biomolecules such as proteins, nucleic acids, fats, cell membranes, tissues, biological fluids, etc., and biosafety to properly use them in biomedical applications. It is universally known that zinc oxide nanoparticles are antibacterial and inhibit the growth of microorganisms by permeating into the cell membrane. The extraction of bioactive chemicals is a crucial step in the synthesis mediated by plants, algae, bacteria, and fungi Nonetheless, the use of microbes as "nanofactories" in contemporary nanotechnology holds significant promise for the synthesis of a wide variety of nanoparticles. These days, the feedstock for the creation of metal nanoparticles is fungi and bacteria. Through the release of physiologically active molecules like enzymes and proteins, this mechanism lowers the concentration of metal ions within nanoparticles. High levels of antifungal activity are exhibited by ZnO-based nanomaterials, which limit the development and reproduction of various pathogenic fungus, including *Macrophomina phaseolina*, *Rhizoctonia solani*, and *Fusarium* sp. Consequently, we report on the manufacture of ZnONPs extracellularly by employing *Trichoderma harzianum*, a putative fungal antagonist. One benefit of mycogenic nanoparticles is that they can produce a capping from fungal biomolecules, which is stable and can support a variety of biological functions [15]. Three monocultures of *Trichoderma* species, including *T. harzianum* and *T. reesei*, were used to create ZnONPs using their fungal secondary metabolites. ZnONPs were biogenically generated with a *T. harzianum* strain cell filtrate as a stabilizer and reducer. In-depth analyses of ZnONP manufacturing techniques, antifungal qualities, and potential antifungal pathways for the control of plant diseases and enhancement of food quality have recently been published. Zinc-based nanoparticles possess targeted antimicrobial capabilities and low to negligible phytotoxic activities, making them effective against a variety of phytopathogens with antibacterial, antifungal, antiviral, and anti-toxigenic properties. Applying these formulations in open fields or greenhouse conditions can be done in several ways [16]. Fungal biomolecules are used to produce a capping that gives stability and can support other biological functions, such as the creation of safe nanofungicides. This is just one advantage of using fungus in the biogenic synthesis of ZnONPs. *T. harzianum* cell filtrate has only been utilized sparingly in publications as a reducer and stabilizer agent during the synthesis of ZnONPs [14, 16]. Consequently**,** our study is aimed at the biosynthesis of ZnONPs to improve sweet pepper (*Capsicum annuum L.*) resistance against *F. oxysporum* and *M. incognita* under greenhouse conditions and their impacts on vegetative growth parameters, and physiological features.

## Materials and methods

The sweet pepper cv. Golden plants selected for collection were obtained from the Arid Lands Cultivation Research Institute greenhouse, located in the City of Scientific Research and Technological Applications in Borg El-Arab, Alexandria, Egypt. It is important to note that this farm is exclusively dedicated to research and development purposes.

### Trichoderma longibrachiatum as bio-agent

*Trichoderma* sp. was kindly obtained from Prof. Dr. Elsayed Belal, Professor of Agricultural Microbiology, Agric. Botany Department, Faculty of Agriculture, Kafr El-Sheikh University according to [17].

### Isolation, purification and identification of pathogenic fungus

Pepper plants showing root rot symptoms were collected from Kafr El-Sheikh governorate at (31°18′N 30°56′E / 31.3°N 30.93°E / 31.3; 30.93), Egypt during the pepper plant season in 2020. Diseased roots were washed with tap water, cut into small pieces and surface sterilized with 0.5% sodium hypochlorite solution for three minutes then washed three times with sterilized distilled water. Samples were dried between two layers of sterilized filter papers to remove the excess water and placed on potato dextrose agar (PDA) medium in *Petri* dishes. Inoculated dishes were incubated at 25 ± 3 ºC for 4-5 days, and the developed fungal cultures were purified using hyphal tip isolation techniques according to [9] the pure cultures were transferred on PDA. Cultural, morphological, microscopical, and phytopathological properties were considered to identify the isolated pathogen. Stock cultures were maintained on PDA slants at 4 ºC for further experiments.

### Pathogenicity test

The *Fusarium oxysporum* pathogenicity test was carried out on pepper plants (cv. Golden). Fusarium was grown in 50 ml glass bottles containing 100 g of barley grains and 50 ml of water previously sterilized in an autoclave at 121 degrees Celsius for 20 minutes. Then, the glass bottles were inoculated with Fusarium discs with a diameter of 5 millimeters taken from a 10-day-old colony, and incubated at 28 °C for two weeks to test their pathogenicity [9]. Three thousand freshly hatched *M. incognita* J2s in 2 mL water were introduced into the mixture and were incubated for 45 days at 27±5°C. Polyethylene bags (15 cm diameter) containing sterilized sandy-clay (1:1 w/w) were inoculated with the pathogens at 2% for ten days before transplanting. All Polyethylene bags were irrigated before planting to ensure a homogeneous distribution of pathogens within the soil. Healthy 30 day-old pepper plant seedlings were transplanted with three seedlings per pot. A control experiment was prepared without the addition of Fusarium. Three pots were used as replicates. The polyethylene bags were placed inside the greenhouse with careful observation. The disease severity index is calculated as stated by [18] according to the following equation.

$$\mathrm{Diseasese}\;\mathrm{verity}\;\mathrm{index}\left(\%\right)=\frac{\Sigma\;\left(rating\;no.\right)\;\left(No.\;plants\;in\;rating\right)}{\left(Total\;no.\;of\;plants\right)\;\left(Highest\;rating\right)}\times100$$

### Identification of the two fungal isolates

To distinguish between the species *Trichoderma* and *Fusarium* isolates, we conducted a growth pattern analysis and observed the morphology of their conidia and conidiophores using stereoscopes. First, a single spore of each species was placed on a PDA plate at a temperature of 25°C for a period of 5 days. Subsequently, a fungal disc with a diameter of 5 mm was placed at the center of the PDA plates (90 mm) and incubated at 25±2 °C in darkness for 7 days. The color, smell, growth, and shape of the colony was examined, as well as the branching pattern of conidiophores and conidia [19, 20]. Following this, a phylogenetic analysis on the 5.8S-ITS region sequence was performed to further identify both Trichoderma and Fusarium isolates. Genomic DNA from each fungus was extracted using PCR amplification mixtures prepared in 0.2ml sterile PCR tubes, with a final volume of 25 µl. The reagents added included 7.5 µl dH2O, 12.5 µl Master Mix (Itron biotechnology—Korea), 1 µl DNA, and 2 µl PCR primer (10 pmol/µl) for each primer. The DNA amplifications were conducted using a thermal cycler (*Eppendorf*, Germany) with the following settings: initial denaturation at 95◦C for 2 min, followed by 34 cycles at 95^◦^C for 40 s, annealing at 56^◦^C for 1 min, extension at 72^◦^C for 1 min, and final extension at 72^◦^C for 10 min. Afterward, the amplification products were stored at 4^◦^C [21]. To analyze the PCR products, electrophoresis was performed on a 1% agarose gel using a current of 100 V for 45 min. The gel was then stained with ethidium bromide and examined under the Gel Documentation System. Finally, the PCR products were purified from the agarose gel using the PCR Clean-Up Column Kit (Maxim Biotech Inc, United States). The DNA Automated Sequencer was used to sequence the PCR products. The sequencing process utilized the same primers used for amplification, and the HiSeq-2000 from Macrogene Scientific Services Company in Korea was employed.

### Construction of Phylogenetic Using the 5.8S-ITS region of the DNA

To establish how closely our fungal isolate's sequences match others, we performed a phylogenetic analysis. After aligning each fungal sequence with those of closely related species, a BLAST search utilizing the NCBI Blast website (https://blast.ncbi.nlm.nih.gov/Blast.cgi) was first performed. We used the CLUSTALW tool in the Bioedit program to further analyze the data. Additionally, we evaluated the similarity of our phylogenetic tree to other fungal species using the BLAST and MEGA 7 tools. The neighbor-joining (NJ) method, as defined by [22]. Was used to build the tree. Based on their great resemblance (more than 96%) to our isolate in a DNA BLAST search, we chose additional fungi for comparison.

### Root knot nematode isolation and morphological identification

Nematode inoculum was extracted from pure cultures of *M. incognita* grown on black nightshade, in the greenhouse of Alexandria City of Scientific Research and Technological Application*.* The extracted galled roots were collected and washed with tap water to remove the adhering soil particles, and the egg masses in the galls were collected with a needle under a stereoscopic microscope (LABOMED; Labo America, Inc. USA). Egg masses were incubated in *Petri* dishes with distilled water for 48h at room temperature (27 ± 2°C) to induce hatching. Active J2s were collected after hatching. Approximately 2000 root-knot nematode J2s were inoculated per pot into one-month-old tomato plants cv. Alisa, which was planted in 2.5 kg of sterile sandy/clay soil mixture in the greenhouse at 27±2°C [23]. After Thirty-five days of nematode inoculation, nematode eggs were extracted from galled roots by washing and cutting the roots into 1 cm pieces, then shaking the root pieces for 3 min in 1 L of 0.5% of sodium hypochlorite solution (NaOCl) [24]. The resulting egg suspension was sieved through 200 and 500 mesh sieves. In 100 ml plastic beakers, nematode eggs retained by the 500 mesh sieve were collected. Nematode eggs were then left to hatch in sterile distilled water at 26±3°C, and newly hatched J2s were collected. Freshly hatched J2s collected were used as nematode inoculum. Ten mature *Meloidogyne* sp*.* females were removed from the root tissue using forceps. Females were separated from egg masses and placed in a drop of warm lactophenol on a clear glass slide to be examined under a light microscope for perennial pattern identification [25].

### Efficacy of *Trichoderma* sp. as a bio-agent against *F. oxysporum*

This strain of *Trichoderma* sp. was selected because it showed great antagonistic capacity against the soil fungus. *Trichoderma* sp. was identified as ITS gene sequencing. The antagonistic effect of the tested biocontrol agents against *F. oxysporum* was examined. A mycelial disc of Trichoderma sp. was placed on PDA plates at 28°C for 24h, and then a mycelial disc of *F. oxysporum* was placed onto PDA plates at 0.5 cm distant from the Trichoderma disc. Three replicates were prepared. Inoculated plates were incubated at 28°C until the fungal growth of the control plates reached the edge of the plate. The growth and reduction in mycelial growth of the pathogenic fungus was calculated as mentioned by [26]. Radial growth of *Trichoderma* sp. and *F. oxysporum* was recorded. Inhibition percent of growth was calculated using the following formula: Growth reduction (%) = X 100 according to [27].

### Mycosynthesis of Zinc Oxide Nanoparticles

*Trichoderma longibrachiatum* was inoculated into 500 mL flasks containing 250mL of PDB medium supplemented with chloramphenicol antibiotics, and incubated in static condition for 20 days at 25±2 ^◦^C. The flask has been shaken well to get a suspension of spores and media which contain secondary metabolites formed because of *T. longibrachiatum* growth. The suspension was filtered through a plastic sieve to remove any remaining solid impurities. Zinc sulfate was used as the precursor the stock solution of zinc sulfate was prepared by dissolving 1.61 g of zinc sulfate in 20 ml of sterilized distilled water to get the 10 mM concentration and used freshly [28] with minor modifications. One mL solution of zinc sulfate was applied per 100 mL fungal suspension and incubated for 5 days at 150 rpm in an orbital shaker. Black precipitate deposition at the bottom of the flask indicated the formation of nanoparticles. After centrifugation at 10000 rpm for 10 min, the pellet aggregated at the bottom of the tube was purified with distilled water and 70% ethanol by repeated centrifugation at 5000 rpm for 20 min. ZnO-NPs were dried overnight at 60 °C in an oven and used for further research. ZnO-NPs characterization was subjected to a variety of instrumental analytical techniques to describe physicochemical properties.

### Characterization of ZnO-NPs

Microscopy technique is an important tool for characterization and imaging of ZnO-NPs. Electron microscopy gives an exact information about the size, structure, shape, spatial resolution, and composition of ZnO-NPs [29]. The following techniques UV–Vis spectrophotometer, SEM , TEM and EDX are used for the morphological identification of ZnO- NPs [30]. Among the listed techniques, SEM and TEM are the techniques commonly used by researchers. ZnO-NPs produced by Trichoderma filtrate were analyzed using an X-ray diffractometer "model (XRD-7000, Shimadzu, Japan) in the City of Scientific Research and Technological Applications lab in Alexandria, Borg EL-Arab, Egypt. Cu-K X-rays of wavelength 1.54060 were assessed, and data were transmitted for 2 at a range of 5° to 80° with a 0.026° step. FT-IR Spectrometer (FTIR-8400S, Shimadzu, Japan) was used to study the chemical structures of zinc nanoparticles. To determine the composition of these zinc nanoparticles, the silver nanoparticles were measured in a range of 400 to 4000 cm-1 using KBr powder.

#### *In-vitro* assessment efficacy of ZnO-NPs against *F.oxysporum* compared with commercial fungicide

*Fusarium oxysporum* was cultured on PDA at 25±3°C. Antifungal tests were performed by the agar dilution method [31] with minor modifications. Before pouring the autoclaved PDA media into the *Petri* dishes (9 cm) placed ZnONPs at concentrations of 4, 6, 8, and 12 mg L^−1^ and an NP-free solution a control. A plug (0.5 cm) of fungal mycelia was taken from the edge of 7 day-old plate, placed in the center of each *Petri* dish, and incubated at 25°C. The efficacy of ZnO-NPs treatment was evaluated by measuring the diameter of fungal colonies and calculate the Inhibition percent of growth. The same technique and concentrations were used to test Uniform 390 SE commercial fungicide effect on *F. oxysporum*. All tests were performed in triplicate and the diameters were expressed in centimeters.

#### Cytotoxicity determination of ZnO-NPs

Normal human lung fibroblast Wi-38 and skin fibroblast HBF4 cell lines were used to detect cytotoxicity of the studied ZnONPs These cell lines were cultured in DMEM medium-contained 10% fetal bovine serum (FBS), seeded as 5x10^3^ cells per well in 96-well cell culture plate and incubated at 37ºC in 5% CO_2_ incubator. After 24 h for cell attachment, serial concentrations of ZnO-NPs were incubated with Wi-38 or HBF4 cells for 72h. Cell viability was assayed by MTT method. Twenty microliters of 5 mg/ml MTT (Sigma, USA) was added to each well and the plate was incubated at 37ºC for 3h. Then MTT solution was removed, 100 µl DMSO was added, and the absorbance of each well was measured with a microplate reader (BMG LabTech, Germany) at 570 nm. The dose (IC_50_ and EC_100_) values (at 50% and100% cell viability, respectively) of the tested compounds was estimated by the Graphpad Instat software. Furthermore, cellular morphological changes before and after incubation with ZnO-NPs at 0.1 mg/ml were investigated using phase contrast inverted microscope with a digital camera (Olympus, Japan).

#### *In-vitro* assessment efficacy of ZnO-NPs on Second‑stage juvenile mortality

A laboratory experiment was carried out to assess the nematicidal and ZnO-NPs 10 mg /100 mL effect on *M. incognita* J2s mortality. Second-stage juveniles were treated with different concentrations of ZnO-NPs (4, 6, 8 and 12 mg L^−1^) compared with Negative control (*M. incognita* without treatment) and positive control (*M. incognita* + Nemacur 400 EC). The bioassay was conducted in 10-well cell culture plates, with approximately 35 freshly hatched J2s / 1 ml each treatment, each treatment replicated three times. The treatments were as follows:

- a) 12 mL ZnO-NPs +1 ml nematode suspension (eggs for eggs hatchability and freshly hatched J2s for mortality bioassay)
- b) 8 mL ZnO-NPs + 4 mL d.H2O + 1 ml nematode suspension.
- c) 6 mL ZnO-NPs + 6 mL d.H2O+1 ml nematode suspension.
- d) 4 mL ZnO-NPs + 8 mL d.H2O+1 ml nematode suspension.
- e) 12 ml distilled H_2_O 1 ml nematode suspension (Negative control).

- f) 12 ml distilled H_2_O + 1 ml nematode suspension +5 μl Nemacur 400 EC(Positive control) .

The plates were incubated at 25 ±2°C for 6,12, 24,72 h and 7 days after treatment, and the mortality of J2s was recorded. The nematodes were considered dead if they appeared motionless in plain water. The percentage of mortality was calculated according to [32].

$$\mathrm{Mortality\%}=\left[\frac{\left(Total number of alive J2 Sin control-No.ofalive J2 Sintreatment\right)}{2a No.of total alive J2 Sin control}\right]\times 100$$

### Greenhouse experiments

#### Inoculum preparation

Inocula of *F. oxysporum* were prepared by growing them on PDB media and incubated at 25±3^◦^C for 10 days. Spore suspensions of the isolate was counted and adjusted to 2 × 10^6^ spore mL^−1^ [33] with slight changes. Three thousand freshly hatched *M. incognita* J2s in 2 mL water were introduced into the mixture and were incubated for 45 days at 27±5°C. The experiment was carried out with ten replicates for each treatment, and the experiment was repeated twice. The most effective treatments were applied against *F. oxysporum* and *M. incognita* on pepper plants cv. Golden. Pot experiment was designed under greenhouse conditions using plastic pots (15 cm) containing autoclaved sterilized sandy loam soil (1:1) infested with one of the two pathogens, *F. oxysporum* or *M. incognita* via three deep holes. Seedlings of 60 day-old, grown in disinfested soil, were transplanted in dark polyethylene bags (15cm in diameter filled with 3kgm of soil).

ZnO-NPs were applied at the time of seedling cultivation as a follow:

1. T1: Seedling without any pathogen (healthy control).
2. T2: Seedling + *M. incognita* (negative control).
3. T3: Seedling + *F. oxysporum* (negative control).
4. T4: (Seedling + *M. incognita* +* F. oxysporum*).
5. T5: Seedling + *M. incognita* + Uniform 390 SE
6. T6: Seedling + *F. oxysporum* + Uniform 390 SE.
7. T7: Seedling + *M. incognita* +* F. oxysporum* + Nemacur + Uniform 390 SE.
8. T8: Seedling + *F. oxysporum* + 12 mL ZnONPs (S1).
9. T9: Seedling + *F. oxysporum* + 8 mL ZnONPs (S2).
10. T10: Seedling + *M. incognita* + 12 mL ZnONPs (S1).
11. T11: Seedling + *M. incognita* + 8 mL ZnONPs (S2).
12. T12: Seedling + *M. incognita* +* F. oxysporum* + 12 mL ZnONPs (S1).

All treatments were administered by drenching the soil with 100 mL of water / pot immediately after inoculation. The pepper plants were kept in the greenhouse under the same conditions as previously described, using a randomized complete block design with 10 cm spacing between each Polyethylene bag for a period of 90 days, greenhouse temperature during the experiment ranged from 25 to 30 °C, and there were ten replicates (pots) where the experiment was conducted twice. After 90 days, the plants were harvested, and their roots were thoroughly washed with tap water to remove any surrounding soil. The following parameters were measured: fresh and dry weights of both roots and shoots, leave area of each plant. To assess the infective ability of *M. incognita*, the roots inoculated with this nematode were soaked in a solution containing 0.015% phloxine B for 15 minutes to stain the egg masses before counting them. The eggs of *M. incognita* were then extracted from the pepper roots using a method provided by [34] and counted under stereoscopy. The number of egg masses was considered an indicator of nematode infectivity, as it represents the number of J2s that successfully penetrated and infected the root tissue to develop into egg-laying females. Additionally, the number of galls and egg masses per root, as well as females per root, were calculated. Furthermore, J2s present in 250 g of soil per pot were extracted and recorded [21]. Total leaf protein concentration was measured according to [35] in two leaf discs (0.8 cm diameter) per plant of each treatment. The results were expressed as µg protein/cm^-2^. The chlorophyll content was measured as an indicator of the plant photosynthetic performance. For the chlorophyll measurement, 0.5 gm of fresh samples were ground with 5 mL of 80% acetone in addition to 0.1% and/or 0.1% CaCO3 to prevent chlorophyllase activities. After grinding, the samples were filtered, and the final volume (25 mL) was shifted to new tubes and characterized using a dual-beam spectrophotometer at A663.2 and A646.8, against the blank (80% acetone) and calculated chlorophyll a ,b and total chlorophyll according to next equations The results were expressed as mg chlorophyll/ g^–1^ [36].

$$\begin{array}{ccc}\mathrm{Chl``a"}=\left(12.25\ast\mathrm{A}663.2\right)-\left(2.79\ast\mathrm{A}646.8\right)\\\mathrm{Chl``b"}=\left(21.50\ast\mathrm{A}646.8\right)-\left(5.10\ast\mathrm{A}663.2\right)\\\mathrm{Total}\,\mathrm{Chl.}=\mathrm{Chl``a"}+\mathrm{Chl``b"}\end{array}$$

At the end of the experiment, up regulated and down regulated genes were determined by Differential Display PCR (DD-PCR) technique with three primers (1, 2, and 3). In addition evaluation of the expression of three oxidative genes (Chitinas, Super Oxide Dismutase, and Ascorpat Peroxidase), by the quantification of gene expression RNA in the pepper root tissue, using Quantitative Poly Chain Reaction (Q-PCR). The roots were washed three times with fresh sterilized distilled water for ten seconds each and then dried on sterile tissue paper. Total RNA was extracted from pepper root samples that were infected with *M. incognita* or *F. oxysporum* and treated with ZnO-NPs at a concentration of 100% (S) at various time points after inoculation with two pathogens (6, 12, 24, 72, day and 7 week after inoculam). TRIzol reagent was used for RNA extraction according to the manufacturer's protocol. The root tissues were ground into a fine powder in liquid nitrogen using a sterilized porcelain mortar and pestle. Approximately 100 mg of root tissue from each treatment was used for RNA extraction, and the resulting RNA was dissolved in RNAase-free water. The integrity of the RNA was checked using ethidium bromide-stained agarose gel, and the concentration of purified total RNA was determined by measuring absorbance at 260 and 280 nm using Spctrostar Nano. cDNA synthesis was performed using oligo (dT) primer, dNTPs, and Moloney-Murine Leukemia Virus Reverse Transcriptase enzyme (M-MLV RT). The three different primers in (Table 1) were used to scan the mRNA transcribed genes of infected and treated plants. For DD-PCR reaction mixture: (25 μl) consisted of 12.5 μl PCR Master Mix (2X) Promega Corporation, 1 μl (50 pmol) of each oligonucleotide primer, 1 μl DNA template, and 9.5 μl nuclease-free water. The reaction cycles consisted of initial denaturation (95 °C, 2 min), 35 cycles of denaturation (95°Cat 31 min), annealing (55 °C at1 min), extension (72 °C at 1 min), and final extension (72 °C at 10 min). The PCR products were analyzed using agarose gel electrophoresis with ethidium bromide (EtBr) staining. As well as The RT-PCR amplification was carried out in a thermal cycler at 40 °C for one hour. Then, it was incubated at 70°C for ten minutes, followed by storage at − 20°C until used. Q-PCR was carried out on the resulting cDNA using a Light Cycler Rotor-Gene 6000 system instrument (Qiagen, United States) with a set of gene-specific primers. Chitinase (Chi), Ascorbate Peroxidase (APX), Superoxide dismutase (SOD), and the pepper ITS 1-4 gene was used as internal (Reference gene) controls as listed in (Table 2). The final volume of each reaction 25 μL consisted of 12.5 μL of 2×Quantitech SYBRGreen RT Mix, forward and reverse primers at 1.5 μL of 10 pmol/μL, 2 μL of template cDNA (50 ng), and 7.5 μL of RNase free water. Reactions were performed in pairs using the following thermal cycling conditions: initial denaturation step at 95 °C for ten minutes. The real-time PCR program included pre-denaturation at 95°C for 20 min before applying 45 cycles of 9 °C for 15s, 60°C for 30s, and then final extension at 72°C for 30s. Relative gene expression data were calculated based on the following equations: ΔC q=C q – reference gene, ΔΔC q=C q – control, and ΔΔC q expression=2 (−ΔΔCq), [37]. Three biological replicates were analyzed.

**Table 1:** Sequence of gene Primers used in DD-PCR

Primer name	Sequence
PPO	5ʹ CACCATGTTGAGCTTCTTCG 3 ʹ
PR1	5' TTCTTCCCTCGAAAGCTCAA 3'
PR3	3' GCGGATCCCAACGCACTGCAACCGATTAT 5'

**Table 2:** The sequence of gene-specific primers, Chitinase (Chi), SuperOxide Dismutase (SOD) and Ascorbate Peroxidase (APX) used in the real-time PCR and ITS gene was used as reference gene

**Primer name**	**Forward primer (5′–3′)**	**Reverse primer (5′–3′)**
Chitinase (Chi)	AATGATGCCGCTTGTCCTG	TCCATAACCCGGTAATCTCCC
SuperOxide Dismutase (SOD)	CCAAATGCCTCGTCATCT	ATTAGAGTCAAGCTCAAAAGG
Ascorbate Peroxidase(APX)	CATTGATAAGGCCAGGAGGA	TTGTTAGCAGCATGACCCTG
ITS1-4	TCCGTAGGTGAACCTGCGG	TCCTCCGCTTATTGATATGC

### Statistical analysis

Data were analyzed by one-way analysis of variance (ANOVA), using Statistical Package (CO-STATE). Duncan's multiple range test at *P* value 0.05, was used to provide significance levels for the difference among the treatment means [38].

## Results

### Isolation of the pathogenic fungus

Isolation trials, carried out on wilt disease, collected from different locations at Kafr El-Sheikh Governorate, Egypt, resulted in the isolation of a fungus isolate belonging to the genus Fusarium as shown by preliminary microscopic examination. The isolated Fusarium isolate was identified as Fusarium sp., which represents infected vessels in the root of the pepper plant compared with an untreated plant. This fungus was previously reported to be associated with pepper wilt disease.

### Identification of *Trichoderma longibrachiatum *and *Fusarium* sp. using rRNA genes

**Fig. 1:**
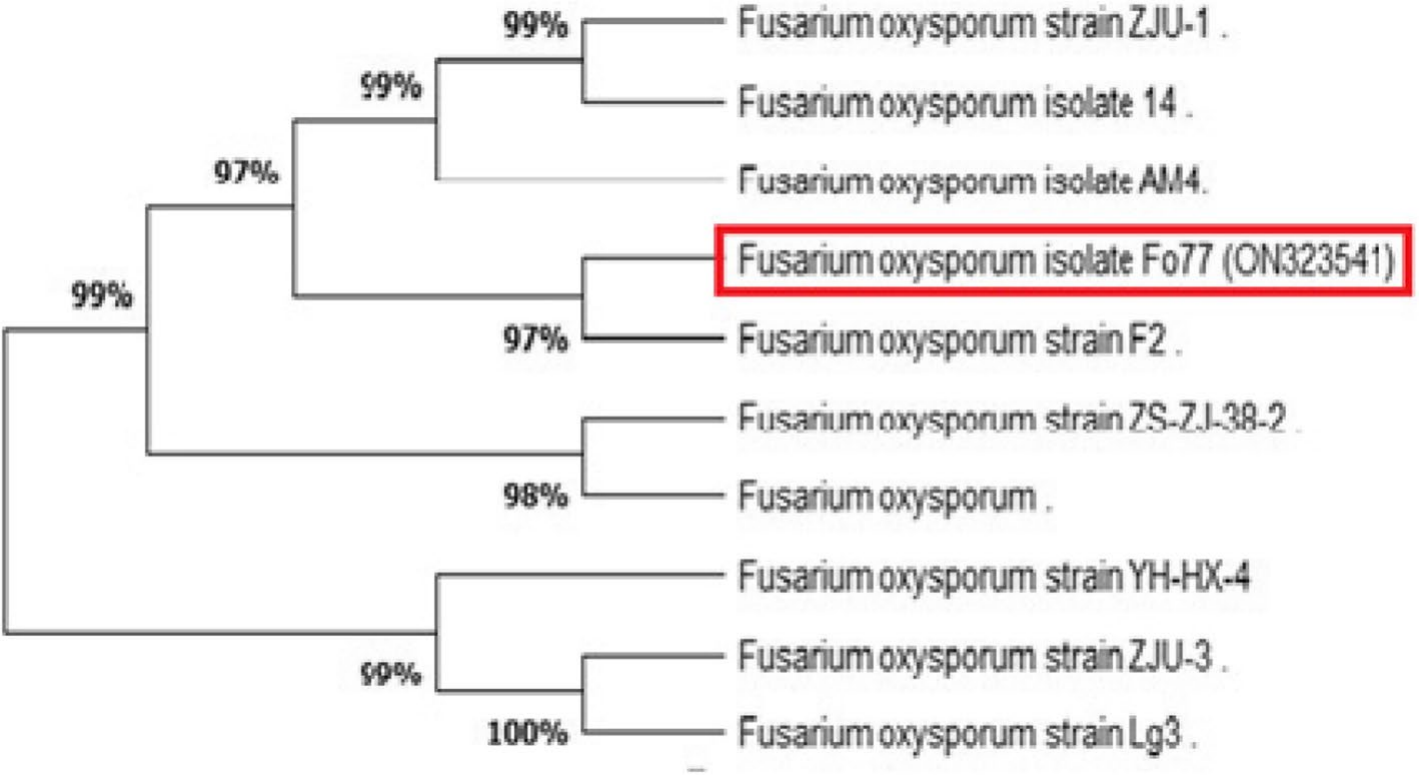
Phylogenetic analysis of *Trichoderma longibrachiatum* based on the gene sequences showing the relationship of *Trichoderma longibrachiatum* to another *Trichoderma* spp. The phylogenetic tree was constructed based on internal transcribed spacer sequences using the maximum parsimony method on MEGA ver. 5.

**Fig. 2:**
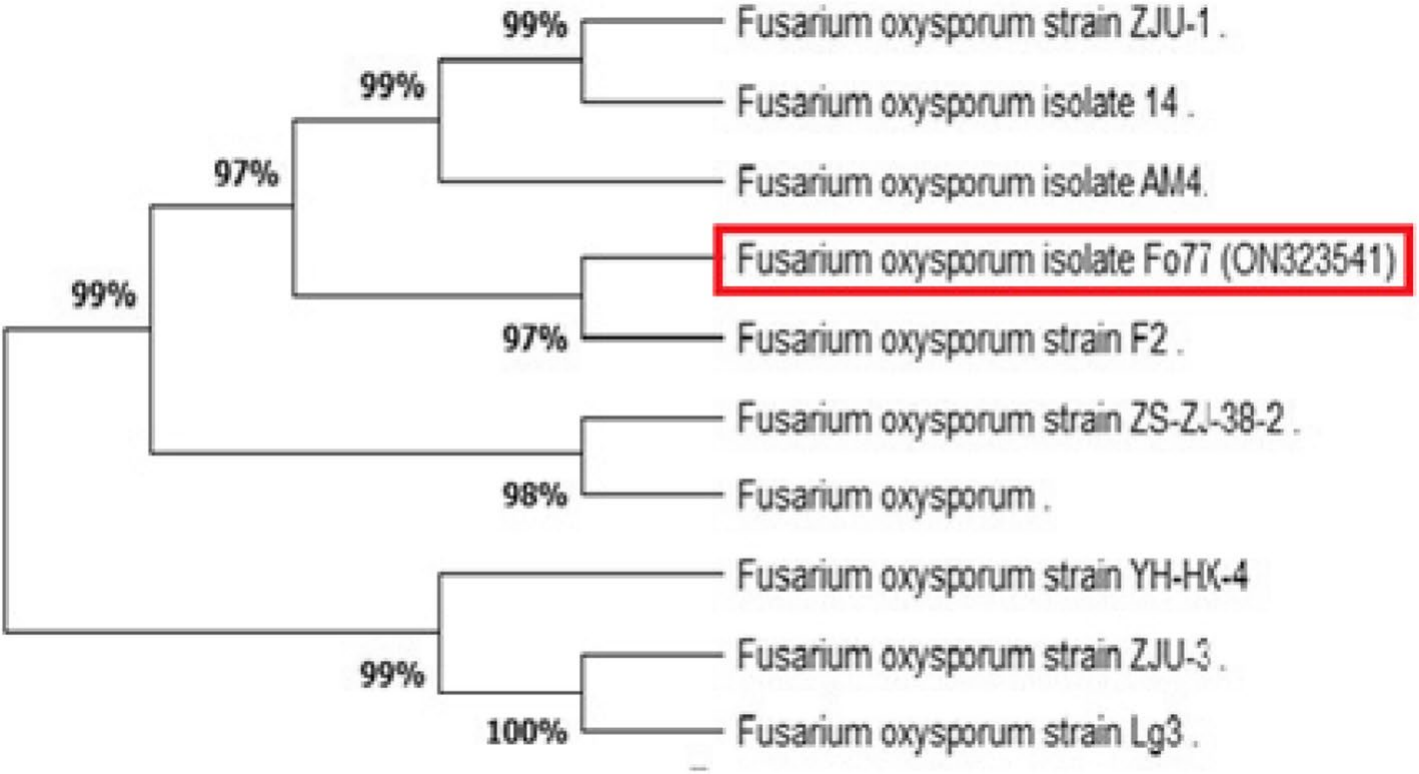
Phylogenetic analysis of *Fusarium oxysporum* based on showing the relationship of *Fusarium oxysporum* to another *Fusarium* spp. The phylogenetic tree was constructed based on internal transcribed spacer sequences using the maximum parsimony method on MEGA ver. 5.

Preliminary identification of fungi was performed based on morphological parameters such as color, spore shape. Amplification and sequencing of fungal rRNA genes resulted in 516 and 504 bp long nucleotide sequence, which have been introduced in NCBI GenBank (Accession Numbers: ON203115 and ON323541p) for *Trichoderma longibrachiatum* and *Fusarium oxysporum*, respectively. The phylogenetic tree exhibited that the *T. longibrachiatum isolate T l T1* was strongly related to the species *longibrachiatum* (Figure 1). On the other hand, it showed the relations between *Fusarium oxysporum* isolate* Fo77* and the other species (Figure 2) The phylogenetic tree was generated using the maximum parsimony method and MEGA ver. 5.

### Disease assessment

The obtained results in (Table 3) revealed that the effect of ZnoNPs, Uniform and Nemacur on the inhibition of *F. oxysporum* and *M. incognita* incidence of pepper plants in pots experiment.

**Table 3:** The effect of treatments on the inhibition of *F. oxysporum* and *M. incognita* incidence of pepper plants in pots experiment

Treatments	Disease severity			
	30 days	45 days	60 days	90 days
Con. (un-inoculated plant)	0 ^g^	0 ^h^	0 ^i^	0 ^i^
Con. *M. incognita*	21.9 ^b^	31.56 ^b^	57.23 ^b^	57.23 ^b^
Con. *F. oxysporum*	20.63 ^bc^	29.33 ^c^	55.46 ^c^	55.46 ^c^
Con. *M. incognita* +* F. oxysporum*	31.13 ^a^	50.1 ^a^	80.53 ^a^	80.53 ^a^
Con. *M. incognita* + Nemacur	0^g^	0 ^h^	0 ^i^	0 ^i^
Con. *F. oxysporum* + Uniform	0 ^g^	0 ^h^	0 ^i^	0 ^i^
Con. *M. incognita*+ Con.* F. oxysporum* +Nemacur+ Uniform	9.3 ^f^	11.33 ^g^	15.2 ^h^	15.26 ^h^
*F. oxysporum* +ZnoNps (S 1)	0 ^g^	0 ^h^	0 ^i^	0 ^i^
*F. oxysporum* +ZnoNps (S 2)	16.83 ^d^	26.06 ^e^	34.73 ^f^	34.73 ^f^
*M. incognita* +ZnoNps (S 1)	11 ^e^	14.46 ^f^	21.1 ^g^	21.1 ^g^
*M. incognita* +ZnoNps (S 2)	17.86 ^d^	27.53 ^d^	36.53 ^e^	36.53 ^e^
*F. oxysporum* + *M. incognita* +ZnoNps (S 1	20.43 ^c^	28.26 ^cd^	46.7 ^d^	46.7 ^d^
LSD 0.05	1.33	1.41	1.08	1.80

Data are mean of three replicates, data with the same letter, in column, are not significantly different at *p*=0.05.

### Optical analysis of ZnO-NPs formation

#### UV–Vis spectral analysis of ZnO-NPs

**Fig. 3:**
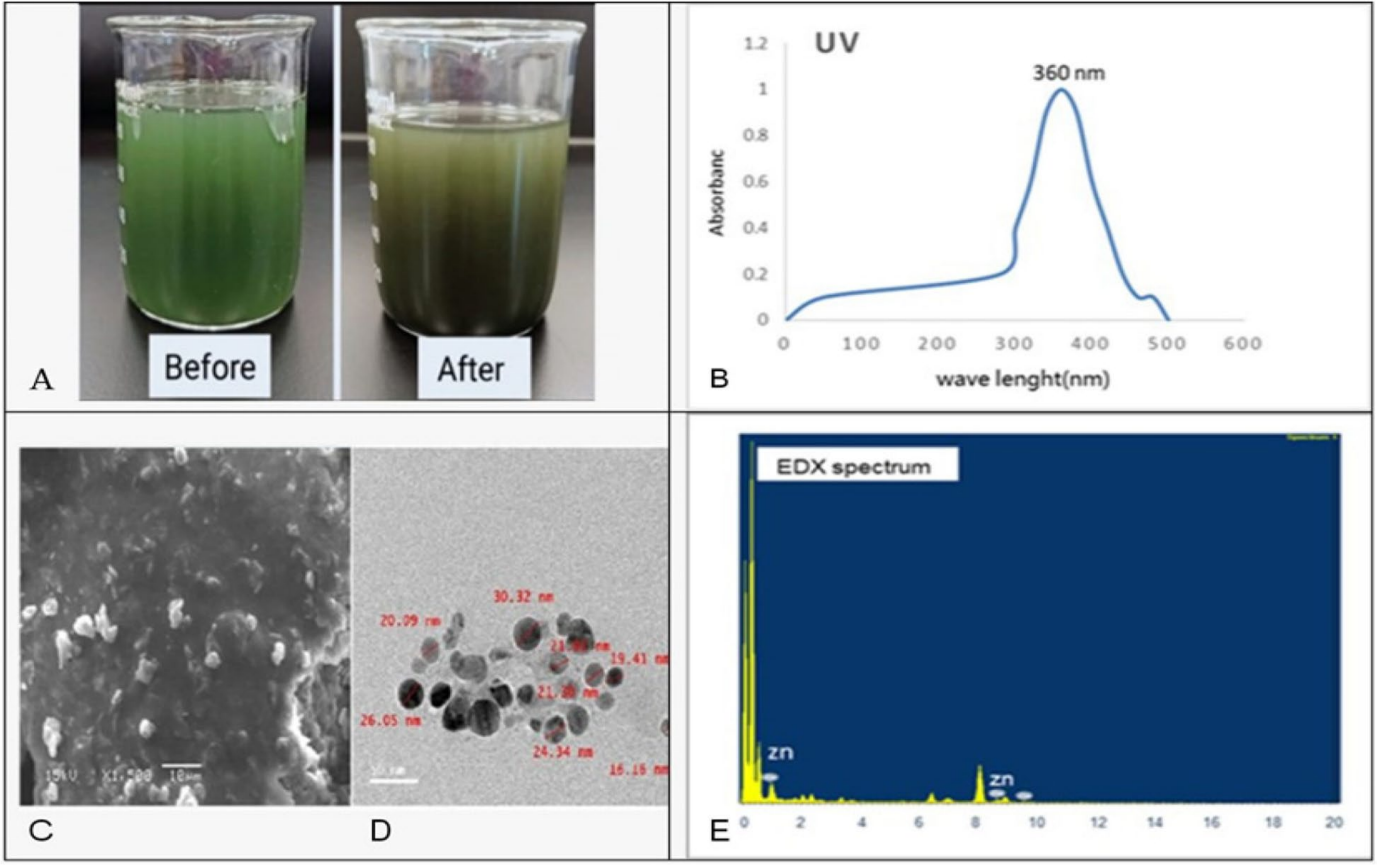
(**A**) change of color from green to dark green indicated the formation of ZnO-NPs. **B** UV-visible spectrophotometer of ZnO-NPs. **C** SEM micrograph of ZnO-NPs showing their morphology at 10µm resolution (**D**) TEM micrograph (**E**) EDX spectrum

The myco-biosynthesis of ZnO-NPs has obtained seriousness suggested as a possible surrogate for physical and chemical methods. Metabolites secreted by Trichoderma have succeeded in the formation of ZnO-NPs, as well as stabilizing these formed nanoparticles. It is well-established that zinc nanoparticles are discernible in brown color. In our present study, zinc nanoparticles were clarified by exposure of filtrate of Trichoderma fungal suspension to the zinc sulfate solution. In aqueous solutions, zinc nanoparticles showed clear green to dark green colors by stimulation of surface plasmon vibrations within the particles. The entire reduction of Zn ions was palpable at 15h incubation. After 5 hours, the color variation was visually observable in the Trichoderma filtrate while incubated with zinc sulfate solution in a covered flask Figure 3(A). Characterization of Zinc nanoparticles. The formation of Zinc nanoparticles was confirmed by visual assessment. The UV–vis spectra of zinc nanoparticles bio/ myco-synthesized by *Trichoderma* filtrate suspension was clarified in Figure 3(B), shows absorption peaks at 360 nm.

#### Scanning electron microscope (SEM)

To study the morphologies of biosynthesized ZnO-NPs, the SEM analysis of colloidal spherical agglomerates of crystallites from Zn-NPs were noted, and ZnO-NPs were observed as deposition on Trichoderma suspension Figure 3(C), the morphology and size details of the formed biosynthesized nanoparticles from Trichoderma suspension**.** Filtrate was characterized and represented by the Transmission Electron Microscope (TEM) micrograph of zinc nanoparticles Figure 3 (D). It is evident from the micrograph that individual zinc nanoparticles, as well as several aggregates, are present and they are spherical with the maximum diameter sizes of 15–30.32 nm for those prepared from the Trichoderma suspension filtrate.

#### Energy dispersive X-ray absorption (EDX)

The EDX spectrum showed clear signals related to zinc in (1) and (9) Kev as shown in Figure 3(E) and signal related to oxygen before (1) Kev, which confirms that the synthesized nanoparticles are principally made of zinc and oxygen.

#### XRD analysis

**Fig. 4:**
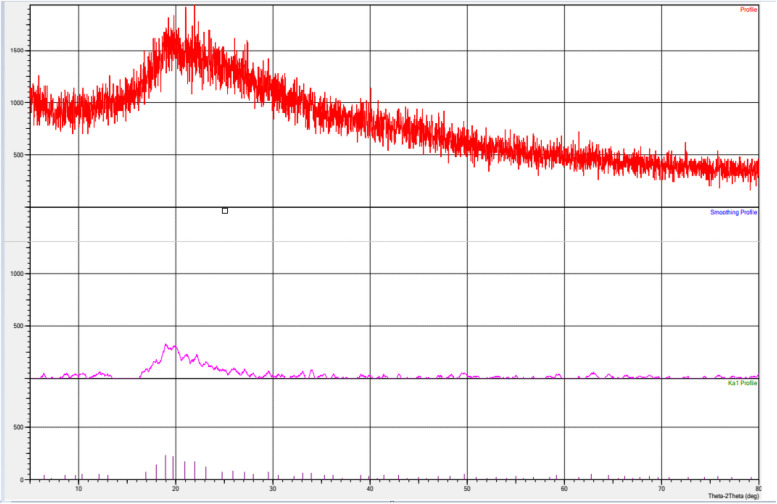
XRD pattern of ZnONPs syntheses by *T. longibrachiatum* filtrate

The diffraction peaks observed in this study can be attributed to polycrystalline ZnO with a hexagonal wurtzite structure. Specifically, three prominent peaks at angles 2θ = 18.94°, 19.72°, and 20.94° correspond to the (100), (97), and (74) planes, respectively. These angles closely match those found in the standard ZnO XRD pattern (JCPDS 89-7102). Notably, no characteristic peaks associated with impurities such as metallic Zn and Zn(OH)2 were detected, indicating the high purity of the synthesized products (Figure 4).

#### FT-IR analysis

**Fig. 5:**
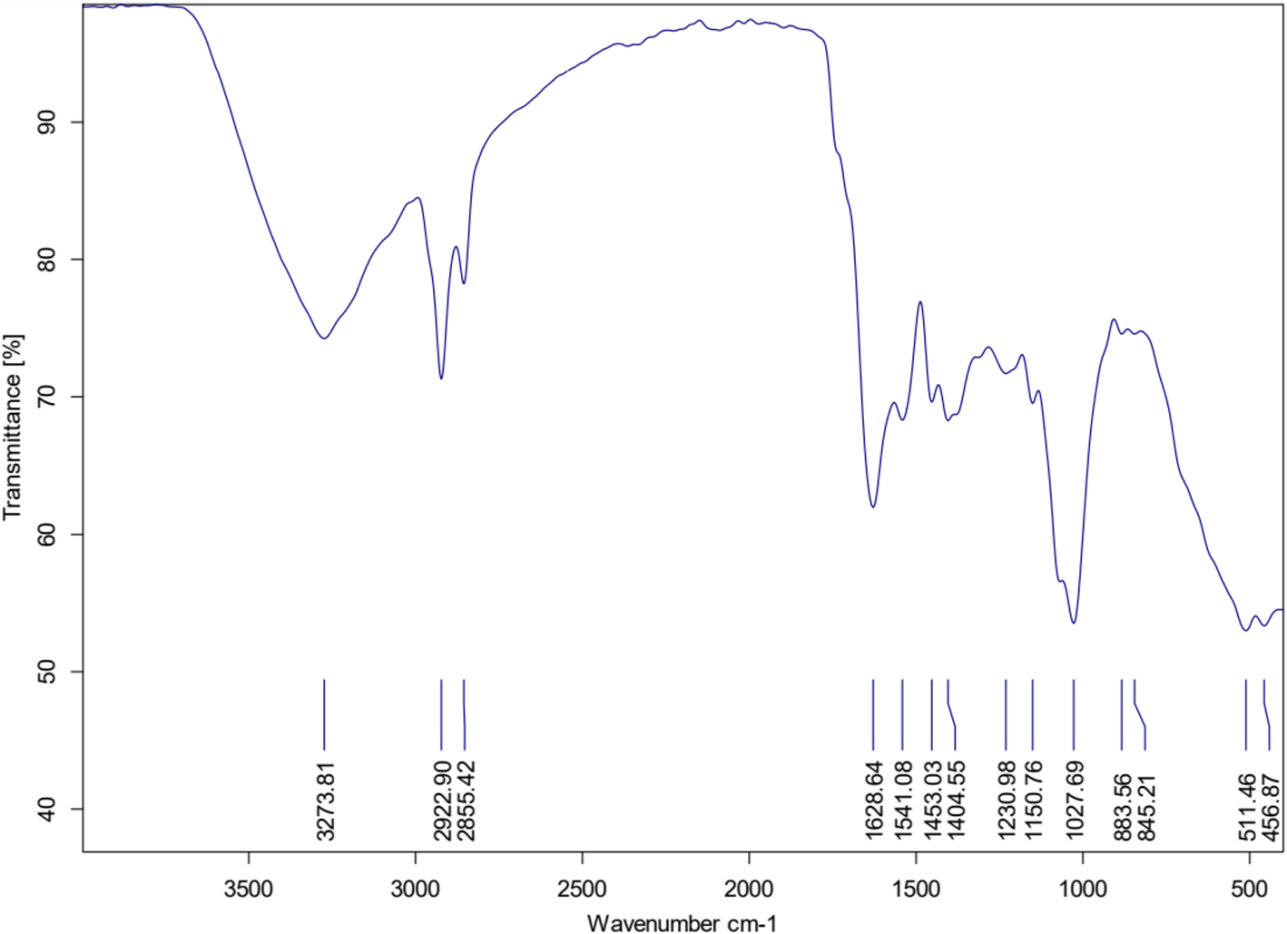
FT-IR spectral pattern of myco-synthesized ZnO-NPs using *T. longibrachiatum* filtrate ranging within 400–3500 cm^-1^.T% Transmittance%

Fourier transform infrared spectroscopy (FT-IR) is a technique used to analyze compounds and identify their functional groups. Figure 5 displays the FT-IR spectrum of phyto-fabricated ZnO NPs, covering a wave number range of 400 to 3500 cm^−1^. In this spectrum, a broad peak at 3273 cm^−1^ indicates the presence of O-H or N-H stretching vibrations from carboxylic acid and phenolic groups. Peaks at 2922 cm^−1^ suggest C–H stretching vibrations, indicating the existence of alkanes associated with the secondary amine ghareeb 2020. Another peak at 2855 cm^−1^ confirms C–H stretching. The presence of C=O due to an amide group is indicated by peaks at 1628 cm^−1^, while another C=O from another amide group is shown at 1541 cm^−1^. Peaks at 1453, 1404, 1230, and 1150 cm^−1^ suggest the occurrence of a C-O group resulting from esters and ethers. The aromatic ether group is represented by the peak at 1027 cm^−1^. Peaks at 883 cm^−1^, 845 cm^−1^, and 511 cm^−1^ correspond to C=C stretching in an alkane group, C=C stretching in an aromatic ring, and stretching in polyphenol (C O), respectively. Additionally, bending vibrations of C–H and stretching vibrations of C–N are indicated by peaks at 456 cm^−1^ (ignorable small peak) and bending vibrations of C–H are indicated by peaks below 500 cm^−1^. These findings confirm the presence of a Zn-O bond as a metal-oxygen bond.

### Antimicrobial efficacy of ZnO-NPs

#### Fungicidal activity of ZnO-NPs against plant pathogenic fungus, *F. oxysporum*

**Fig. 6:**
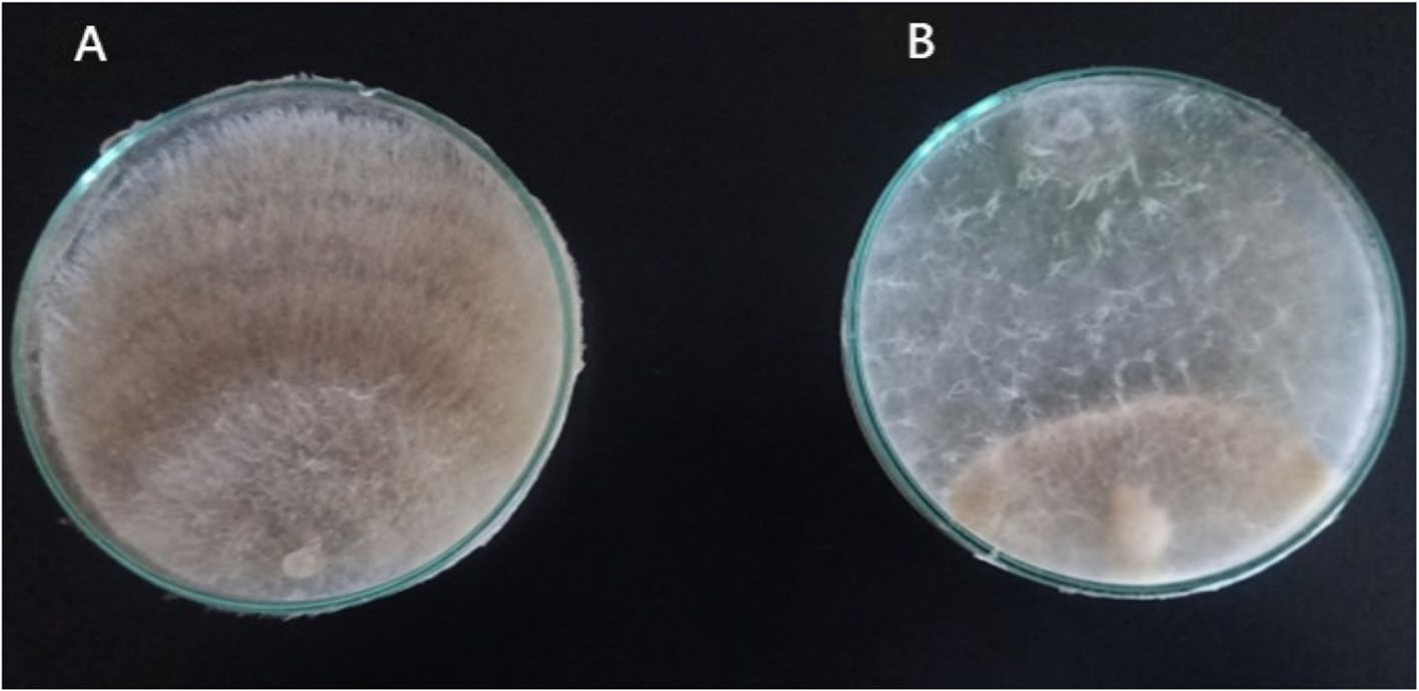
The antagonistic effect of *Trichoderma longibrachiatum* against *Fusarium oxysporum*

**Fig. 7:**
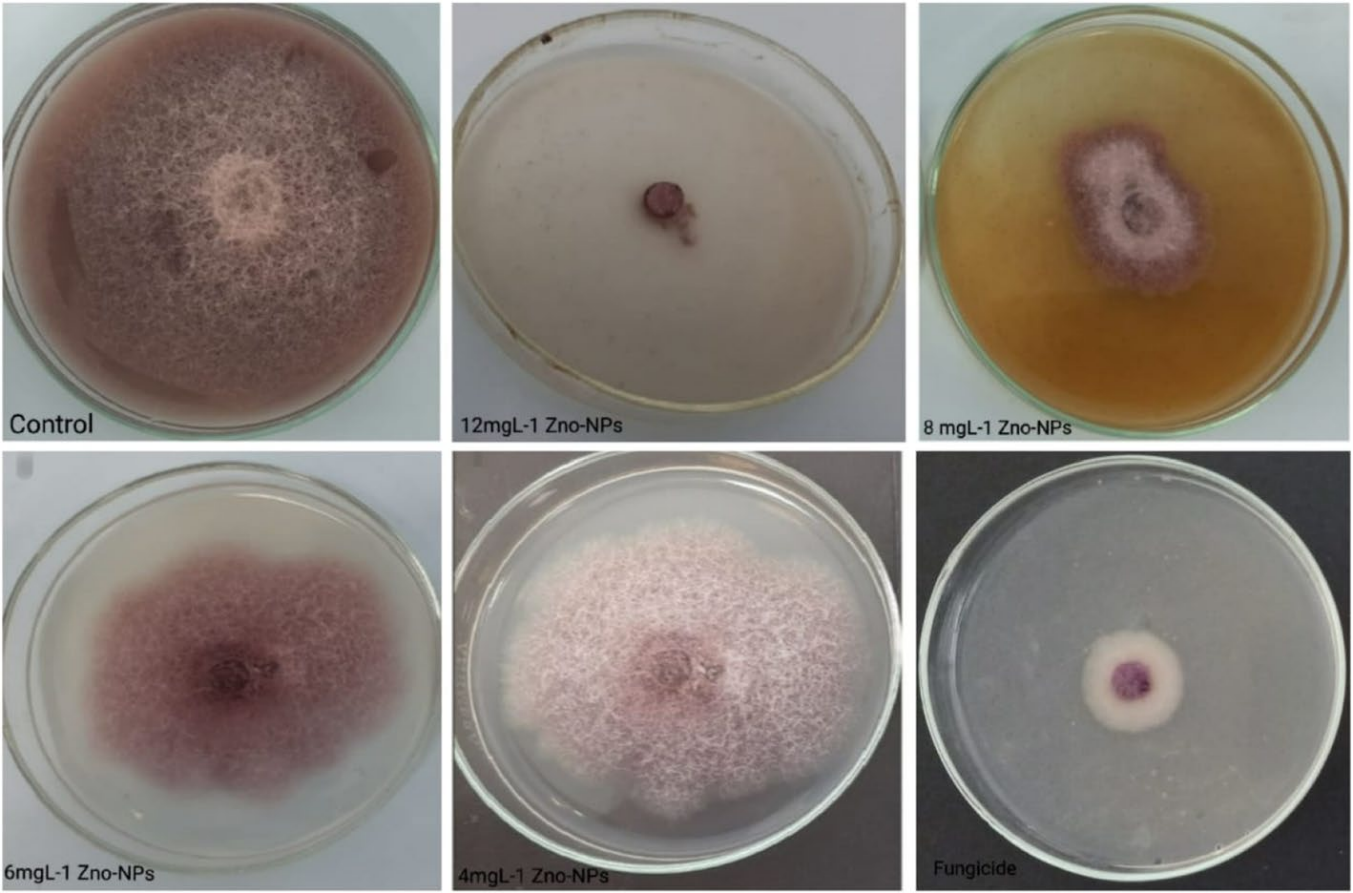
The effect of different concentrations from ZnONPs and fungicide (Uniform) on *Fusarium oxysporum* growth

*Trichoderma longibrachiatum* exhibits antagonistic activity towards *Fusarium oxysporum*, according to Figure 6. The effectiveness of the ZnO-NPs concentrations as inhibitors against the plant pathogenic fungus *F. oxysporum* was also tested. The findings in Table 4 and Figure 7, demonstrated that Uniform 390 SE treatment applications and 12 mg L^-1^ ZnO-NPs had the highest effective inhibition % with 77.03 and 80.73 %, respectively followed by 62.58 % inhibition incase using *T. longibrachiatum*, The lowest inhibition % of *F. oxysporum* was detected with 46 and 56 % with 6 and 4 mg L^-1^ ZnO-NPs concentrations, respectively. ZnO-NPs proved that it has more Inhibitory effect on *F. oxysporum* growth than commercial fungicide and *T. longibrachiatum*. According to [39]. ZnO-NPs show a great inhibitory effect on microbial activity because of their unique properties like large surface area [31]. Used SEM analysis to examine *F. oxysporum* structural changes after ZnO-NPs treatment and found that, after treatment with 12 mg l^−1^ ZnO-NPs, hypha lost their smoothness and appeared swollen and crumbled and incase of untreated sample (the control) observed that the hypha was with typical net structure and smooth surface. This result mean that ZnO-NPs inhibitory effect on *F. oxysporum* growth may be due to deformation in the structure of fungal hypha.

**Table 4:** Suppressive effect of ZnO-NPs on tested isolates of *F. oxysporum*

Treatment	Concentration mg L^-1^	Inhibition zone (%)
*T. longibrachiatum*	-	62.58^b^
ZnO-NPs	12	80.73^a^
	8	61.47^b^
	6	56.66^b^
	4	46.66^c^
Uniform 390 SE	-	77.03^a^
LSD 0.05		7.28

^*^Data are mean of three replicates, data with the same letter, in column, are not significantly different at *p*=0.05.

Different letters indicate significant differences between treatments at *p* ≤ 0.05

#### Nematicidal activity of ZnO-NPs on *M. incognita* under *In-vitro* conditions.

The effect of the commercial nematicide, Nemacur and Bio-synthesized ZnO-NPs were tested against the J2s of *M. incognita*. All treatments increased mortality of *M. incognita* compared with the negative control (*M. incognita* + distilled H_2_O without treatment) after 7 days from exposure time. The maximum mortality was recorded by the treatment 12 mg L^−1^ ZnO-NPs by 97.46 % mortality. While it was 11.82%, 37.63%, 40.86% and 89.65% after 6h, 12h, 24h and 72h, respectively. The other treatments were ordered in a descending pattern as follows; MI+ Nemacur, MI+8mg L^-1^ ZNO-NPs, MI+6 mgL^-1^ ZNO NPs, MI+4mg L^-1^ ZNO-NPs and MI+ d. H_2_O, presented in (Table 5). These results proved that ZnONPs can be a strong candidate as eco-nematicide against root-knot nematode *M. incognita*. Biosynthesized ZnO-NPs can produce toxic effects in the* M. incognita* under different exposure times in vitro conditions. Using ZnO-NPs gave high toxicity for *M. incognita* through 72h. There are several studies examined the toxicity of ZnO-NPs towards various pests, especially nematodes.

**Table 5:** The effects of Nemacur and synthesized Zinc oxide nanoparticles on J2s mortality % (M) of *Meloidogyne incognita* (MI) after 6, 12, 24,72 h and 7 days of exposure

Treatment	Exposure time, number of alive J2s (L) and mortality % (M)									
	6h		12h		24h		72h		7 days	
	L	M (%)	L	M (%)	L	M (%)	L	M (%)	L	M (%)
(Neg. control) MI+ d. H_2_O	31^a^	-	31a	-	31^a^	-	29 ^a^	-	26.33^a^	-
(Pos. control) MI + Nemacur	28^ab^	9.67	22.33^b^	34.40	19.66^cd^	36.55	4^cd^	86.2	1.33^d^	94.93
MI+ 12mg L^-1^ ZNO-NPs	27.33^b^	11.82	19.33^b^	37.63	18.33^d^	40.86	3^d^	89.65	0.66^d^	97.46
MI+8mg L^-1^ ZNO-NPs	29.3^ab^	5.37	20.33^b^	27.95	22.33^c^	27.95	8.33^c^	71.26	5.33^c^	79.74
MI+6mg L^-1^ ZNO-NPs	30.33^ab^	2.15	29.66^a^	4.30	26.66^b^	13.97	22.33^b^	22.98	12^b^	54.43
MI+4mg L^-1^ ZNO-NPs	30.6^ab^	1.07	29.66^a^	4.30	27.66^b^	10.75	25.66^ab^	11.49	15.33^b^	41.77
**LSD 0.05**	3.27	-	3.53	-	3.05	-	4.81	-	3.53	-

Data are means of five replicates; means are stated with the same letter(s) in each column and are not significantly different at (*P*≤0.05). (M) mortality (%) =[Total number of incubated eggs – total number of hatched eggs in treatment]/total number of incubated eggs]×100.

Different letters indicate significant differences between treatments at *p* ≤ 0.05

### Greenhouse Experiments

The nematicidal and fungicidal activities of the concentration, 12 and 8 mg-1L of the ZnO-NPs were performed under greenhouse conditions. The nematicidal effects of the most active concentrations of 12 and 8 mg ZnO-NPs showed that all treatments increased the root fresh weight compared to control (Table 6), reduced root galling by 98% and 77%, number of egg masses by 99% and 92.7%, and number of eggs by 99.9% and 84.5% in the case of plants infected with *M. incognita* only. In the same trend, in the case of combination infected between *M. incognita* and *F. oxysprum* with 12 mg reduced root galling by 52%, number of egg masses by 82%, number of eggs by 77.7% and females by 26.6%. Whereas the effect of ZnO-NPs on plant length varied significantly compared with other treatments.

**Table 6:** The effect of Zno-NPs on the numbers of *M. incognita* galls, egg masses, females/root system, and the number of second-stage juveniles (J2s)/250g soil pepper plant infected with *M. incognita* only and combination between *F. oxysporum* after 90 days

**Treatment**	***M. incognita* Nematodes parameters**							
	**Galls**	***R%***	**Eggmasses**	***R%***	**J2s/250 g soil**	***R%***	**Females/Root**	***R%***
Con. (*M. incognita* )	656 ^a^	-	213 ^a^	-	877.8 ^a^	-	9 ^a^	-
Con.(* F. oxysporum*+ *M. incognita* )	67.6 ^d^	89.6	13.8 ^c^	93.5	38 ^d^	95.6	3.8 ^bc^	57.7
Mj +Nemacur	11.4 ^d^	98.2	3.4 ^c^	98.4	2 ^d^	99.7	0.4 ^c^	95.5
Con.N+ Con.F +Nemacur+Uniform	10.6 ^d^	98.3	3.6 ^c^	98.3	1.8 ^d^	99.8	0.2 ^c^	97.7
*M. incognita* +Zn NPs (S 1)	12.8 ^d^	98	2 ^c^	99	3.2 ^d^	99.6	0.4 ^c^	95.5
*M. incognita* +Zn NPs (S2)	150.6 ^c^	77	15.4 ^c^	92.7	135.4 ^c^	84.5	2.6 ^c^	71
*F. oxysporum*+ *M. incognita* +Zn NPs (S1)	315.6 ^b^	51.9	38 ^b^	82.1	195.4 ^b^	77.7	6.6 ^ab^	26.6
**LSD 0.05**	57.49	-	18.13	-	45.71	-	3.36	-

Data are means of ten replicates; means with the same letter(s) in each column are not significantly different at (*P*≤0.05).

**Fig. 8:**
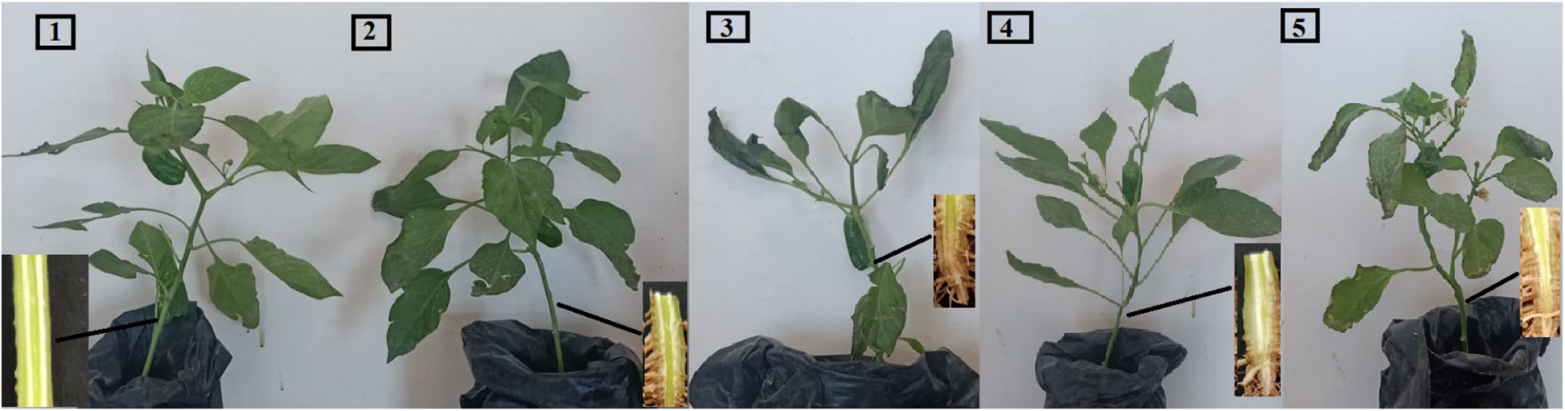
Efficacy of the treatments on the pepper plants (1) control inoculated with *Fusarium oxysporum* (2) control inoculated with *Fusarium oxysporum* and treatment with Uniform (3) inoculated with *Fusarium oxysporum* and treated with Zn-ONPs 100% (4) inoculated with *Fusarium oxysporum* and treated with Zn-ONPs 50%

Different letters indicate significant differences between treatments at *p* ≤ 0.05

(Table 7) showed that the effect of ZnO-NPs on pepper Length, fresh weight, dry weight and leaves area. The data regarding that, plant length showed that ZnO-NPs significantly increased plant length compared to other treatments increasing the highest plant length at 19.5 cm with the treatment of (*F.oxysporum*+12mL ZnO-NPs) followed by 19.16 cm with (*M.incognita*+12 mL ZnO-NPs) compared to 18.28cm with healthy control, treatments 17.92 cm (control *M. incognita* + Nemacur), 17.24cm (control Fusarium+ Uniform) and 16.9cm (control *M. incognita* + control *F. oxysporum* + Nemacur+ Fungicide). The maximum highest plant length for root 9.02cm *F. oxysporum* with 12 mL ZnO-NPs followed by 8.94cm *M*. *incognita* with the same concentration compared to 8.76, 7.96, 7.44 and 6.94cm with healthy control, the control *M. incognita* with Nemacur, control *F. oxysporum* with Uniform and control *M. incognita* + control *F. oxysporum*+ Nemacur + Uniform), respectively. As well as the maximum weight for fresh shoots 6.37g *F. oxysporum* +12mL ZnO-NPs followed by 5.61g with *M. incognita*+ 12mL ZnO-NPs compared to 5.68g healthy control. In addition, the leave area 364 cm^2^ (*F. oxysporum*+12mL ZnO-NPs) followed by 308 cm^2^ *in M. incognita* + 12 mL ZnO-NPs compared to 308 cm^2^ healthy control. According to the results displayed in Figure 8, plants treated with ZnONPs at a 50% concentration showed perfect symptoms of Fusarium infection, while pepper plants treated with ZnONPs at a 100% concentration compared with plants treated with uniform fungicide continued to thrive throughout the experiment, indicating the potential of these ZnONPs to control Fusarium wilt.

**Table 7:** The effect of bio-synthesized Zinc oxide nanoparticles on some growth parameters of pepper plants infected with* F. oxysporum* and *M. incognita* after 90 days in pot experiment

**Treatment**	**Length (cm)**				**Fresh Weight (g)**				**Dry Weight (g)**				**Leafe area (cm2)**	
	**Shoot**	***I %***	**Root**	***I %***	**Shoot**	***I %***	**Root**	***I %***	**Shoot**	***I %***	**Root**	***I %***		***I %***
**Con. (Healthy)**	18.28 ^ab^		8.76 ^abc^		5.68 ^ab^		1.57 ^ab^		1.21 ^abc^		0.33 ^abcd^		308.19 ^ab^	
**Con. *(M.incognita*)**	17.14 ^bcd^	93.	7.3 ^cde^	83	4.64 ^bcd^	81	1.70 ^a^	108	1.15 ^abcd^	95	0.41 ^a^	124	274.67 ^bc^	89
**Con. (*F.oxysporum*)**	17.02 ^bcd^	93	7.8 ^abcd^	89	4.94 ^bcd^	86	1.21 ^bc^	77	1.21 ^abc^	100	0.27 ^de^	81	279.10 ^bc^	90
**Con.(Mj+Fo)**	16.78 ^bcd^	91	6.44 ^de^	73	4.01 ^cd^	70	1.59 ^a^	101	0.90 ^de^	74	0.32 ^bcd^	96	211.24 ^de^	68
**Con.Mj + Nemacur**	17.92 ^abc^	98	7.96 ^abcd^	90	5.27 ^ab^	92	1.52 ^ab^	96	1.09 ^bcd^	90	0.30 ^cde^	90	267.07 ^bcd^	86
**Con.Fo+Fungicide**	17.24 ^bcd^	94	7.44 ^bcde^	84	5.17 ^abc^	91	1.33 ^abc^	84	1.11 ^abcd^	91	0.27 ^de^	81	242.90 ^cde^	78
**Con. Mj+ Con.Fo + Nematicide+Fungicide**	16.98 ^bcd^	92	6.94^de^	79	5.23 ^abc^	92	1.50 ^ab^	95	1.25 ^abc^	103	0.35 ^abcd^	106	223.91 ^cde^	72
**Fo +Zn NPs (S 1)**	19.5 ^a^	106	9.02 ^a^	102	6.378 ^a^	112	1.68 ^a^	107	1.39 ^a^	114	0.37 ^abc^	112	364.07^a^	118
**Fo+Zn NPs (S 2)**	15.8 ^d^	86	5.94 ^e^	67	3.91 ^d^	68	1.09 ^d^	69	0.77 ^e^	63	0.23 ^e^	69%	198.3 ^e^	64
**Mj +Zn NPs (S 1)**	19.16 ^a^	104	8.94 ^ab^	102	5.61 ^ab^	98	1.56 ^ab^	99	1.35 ^abc^	111	0.33 ^abcd^	100%	308.34 ^ab^	100
**Mj +Zn NPs (S 2)**	16.36 ^cd^	89	6.88 ^de^	78	4.45 ^bcd^	78	1.35 ^abc^	85	1.06 ^cd^	87	0.31 ^bcde^	93%	194.45 ^e^	63
**Fo. + Mj. +Zn NPs (S 1)**	16.82 ^bcd^	92	6.58 ^de^	75	5.01 ^bcd^	88	1.7 ^a^	108	1.36 ^ab^	112	0.40 ^ab^	121%	185.55 ^e^	60
**LSD 0.05**	**1.547**		**1.392**		**1.09**		**0.333**		**0.255**		**0.080**		**53.45**	

Data are means of ten replicates; means with the same letter(s) in each column are not significantly different at (*P*≤0.05).

Different letters indicate significant differences between treatments at *p* ≤ 0.05

#### Measurement of Total leaf protein and total chlorophyll

In this study, total leaf protein content and total chlorophyll were measured to find out the impact of ZnONPs compared to controls treatment. The results clarified that, there were significant differences in total protein contents and total chlorophyll among treatments (Table 8). The highest concentration of protein and chlorophyll produced when treating the plant with 12 mg ZnONPs.

**Table 8:** Total protein contents and total chlorophyll in pepper plant chlorophyll produced ring with controls

**Treatment**	**Total Protein**	***Chl.A***	***Chl.B***	***Total chl.***
**Con. (Healthy)**	11.26 ^b^	52.16 ^ab^	61.48 ^ab^	113.64 ^abc^
**Con. *M. incognita***	9.85 ^de^	50.78 ^ab^	46.86 ^cdef^	97.64 ^e^
**Con. *F. oxysporum***	9.89 ^de^	54.41 ^ab^	41.85 ^defg^	96.27^ef^
**Con. *M. incognita* +* F. oxysporum***	9.20 ^e^	41.58 ^b^	33.39 g	74.98 ^g^
**Con. *M. incognita* + Nemacur**	10.89 ^bcd^	52.57 ^ab^	55.88 ^bc^	108.46 ^cde^
**Con. *F. oxysporum* +Uniform**	10.23 ^bcde^	48.97 ^ab^	50.43 ^bcde^	99.41 ^de^
**Con. *M. incognita*+ Con.* F. oxysporum* +Nemacur+ Uniform**	11.14 ^bc^	57.53 ^a^	53.82 ^bcd^	111.35 ^bcd^
***F. oxysporum* +ZnoNPs (S 1)**	12.34 ^a^	55.16 ^ab^	70.70 ^a^	125.86 ^a^
***F. oxysporum* +ZnoNPs (S 2)**	10.14 ^bcde^	48.45 ^ab^	36.33 ^fg^	84.78 ^fg^
***M. incognita* +ZnoNPs (S 1)**	11.18 ^bc^	53.17 ^ab^	70.26 a	123.43^ab^
***M. incognita* +ZnoNPs (S 2)**	10.08 ^cde^	41.81 ^b^	40.64 ^efg^	82.46 ^g^
***F. oxysporum* + *M. incognita* +ZnoNPs (S 1)**	10.19 ^bcde^	43.42 ^ab^	31.87 ^g^	75.29 ^g^
**LSD 0.05**	**1.03**	**12.86**	**11.42**	**12.14**

Different letters indicate significant differences between treatments at *p* ≤ 0.05

### Cytotoxicity assessment of ZnONPs

**Fig. 9:**
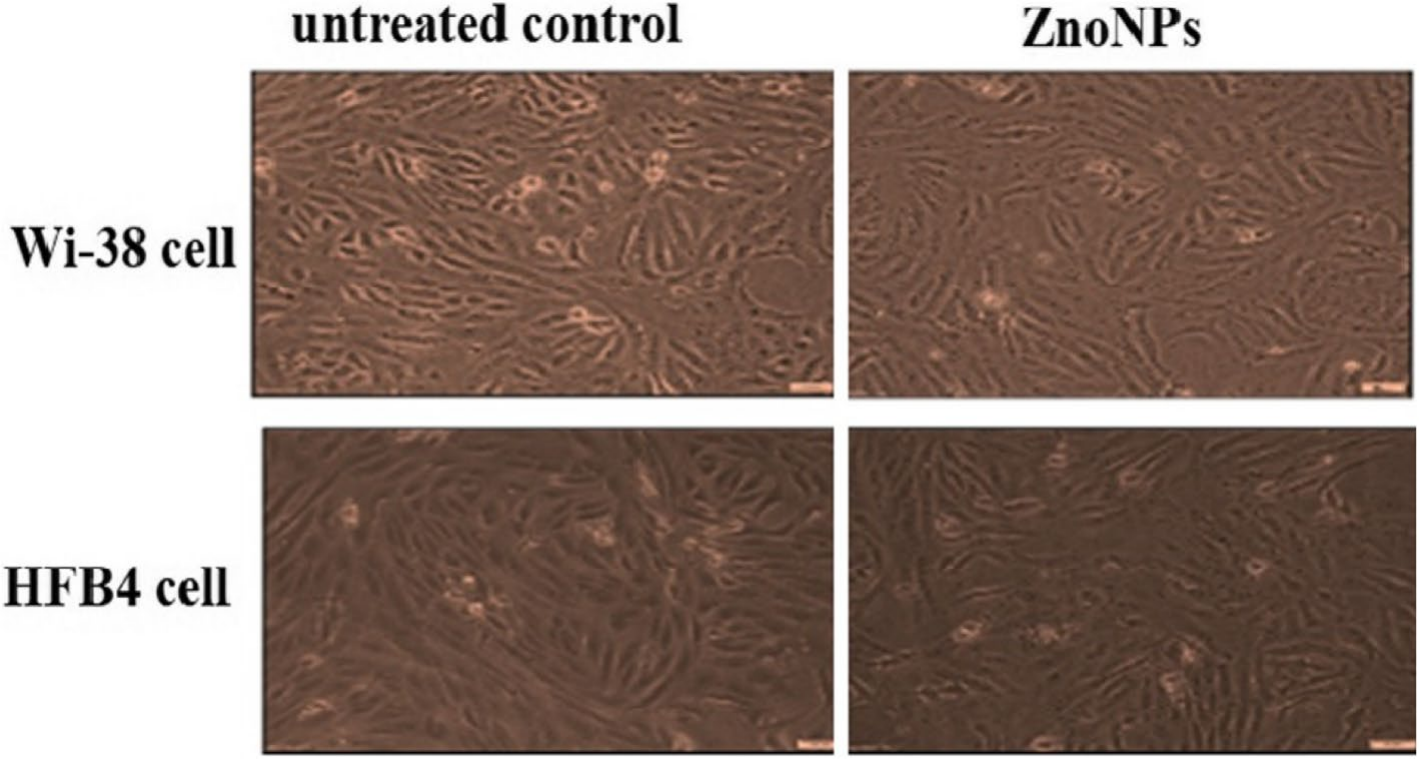
Microscopic photos display the examined ZnONPs compounds safety on both normal cell lines at 0.1mg/ml

Based on the highest EC100 and LC50 values refer to the highest safety on the proliferation of normal human cells (Wi-38 and HBF4, the studied compound safest (Table 9). Accordingly, ZnONPs was the lowest cytotoxicity on both used cell lines. This data was supported by (Figure 9) that illustrated safety of ZnONPs investigated compounds, on both normal cell lines at 0.1mg/ml.

**Table 9:** Cytotoxicity of the tested compounds on Wi-38 cells in the term of EC100 and IC50

Sample	mg/ml	Wi-38		HBF4	
		EC100	IC50	EC100	IC50
Zn	**Mean**	**0.45**	**1.14**	**0.22**	**1.07**
	SEM	0.01	0.09	0.03	0.02

All values are expressed as mean± SEM.

### Differential Display Polymerase Chain Reaction (DD-PCR)

**Fig. 10:**
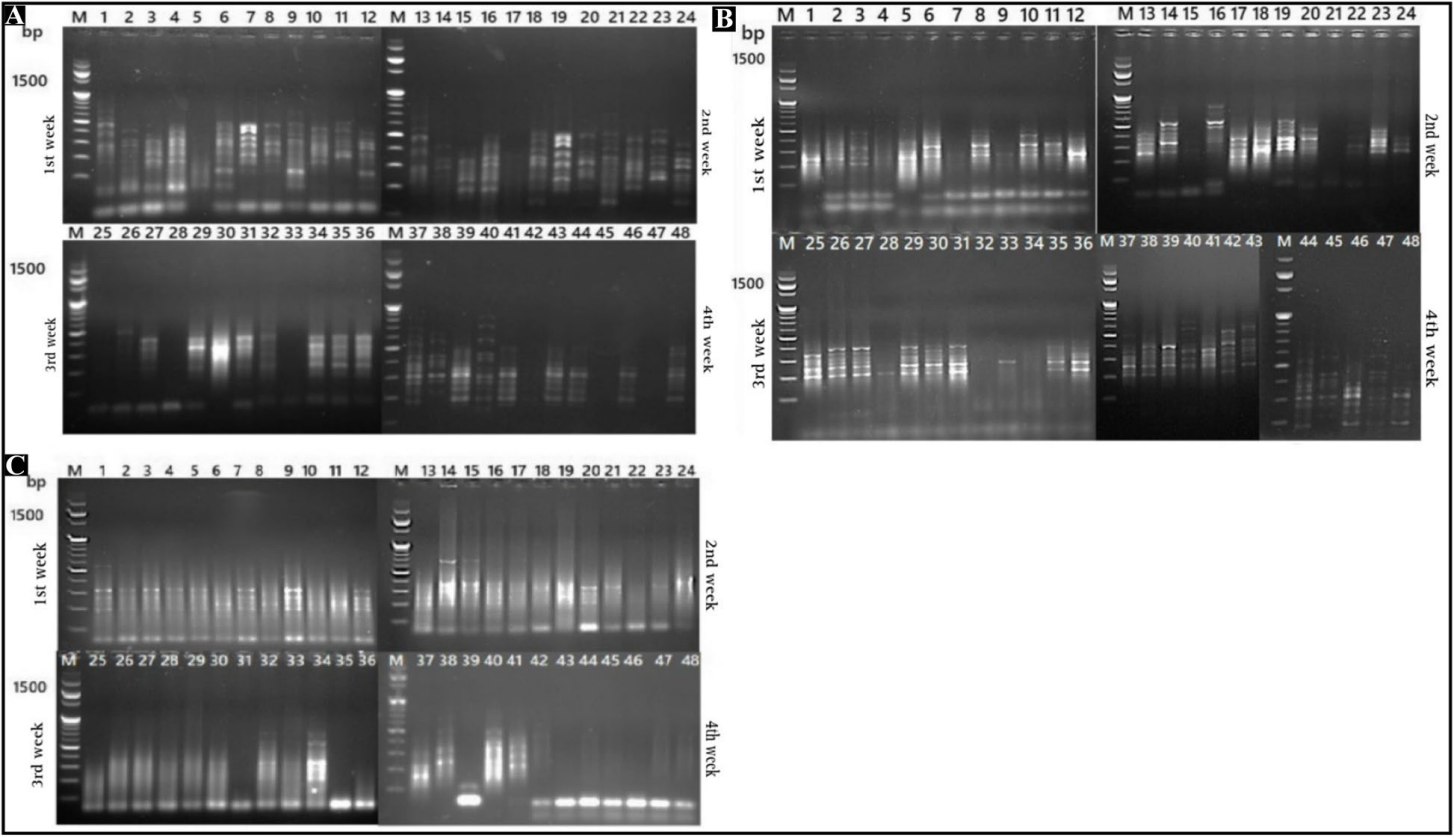
Agarose gel electrophoresis (1.5%) TBE buffer stained with ethidium bromide, showing differential display PCR using (A): PPO, (B): PR1, and (3): PR3 primers. DNA marker 1500 bp; samples 1:12 = 1st week after treatment with ZnO-NPs, (13:24)= 2nd week after, treatment with ZnONPs, (25:36)= 3rd week after treatment with ZnO-NPs, (25:36)= 4th week after treatment with ZnO-NPs

Extracted mRNA from Pepper leaf was used to synthesize cDNA and was subsequently subjected to PCR differential display analysis. The completely differentially expressed genes were carefully investigated through the comparison among all treatments besides healthy control at different time points following treatment with ZnO-NPs. Variation in gene and density was carefully examined in the amplified cDNA in the form of bands detected in the examined plant, up regulated bands that are only present in treated examined samples but absent in the control. The major observation was an increase in genetic variations between controls and plants treated with ZnO-NPs. The results of differential display revealed that many down-regulated (turned off) and up- regulated genes (turned on) were observed in the examined treatments with three primers when compared with control samples, as illustrated in Figure 10 (A, B and C).

### Quantitative Real-time PCR (QPCR)

**Fig. 11:**
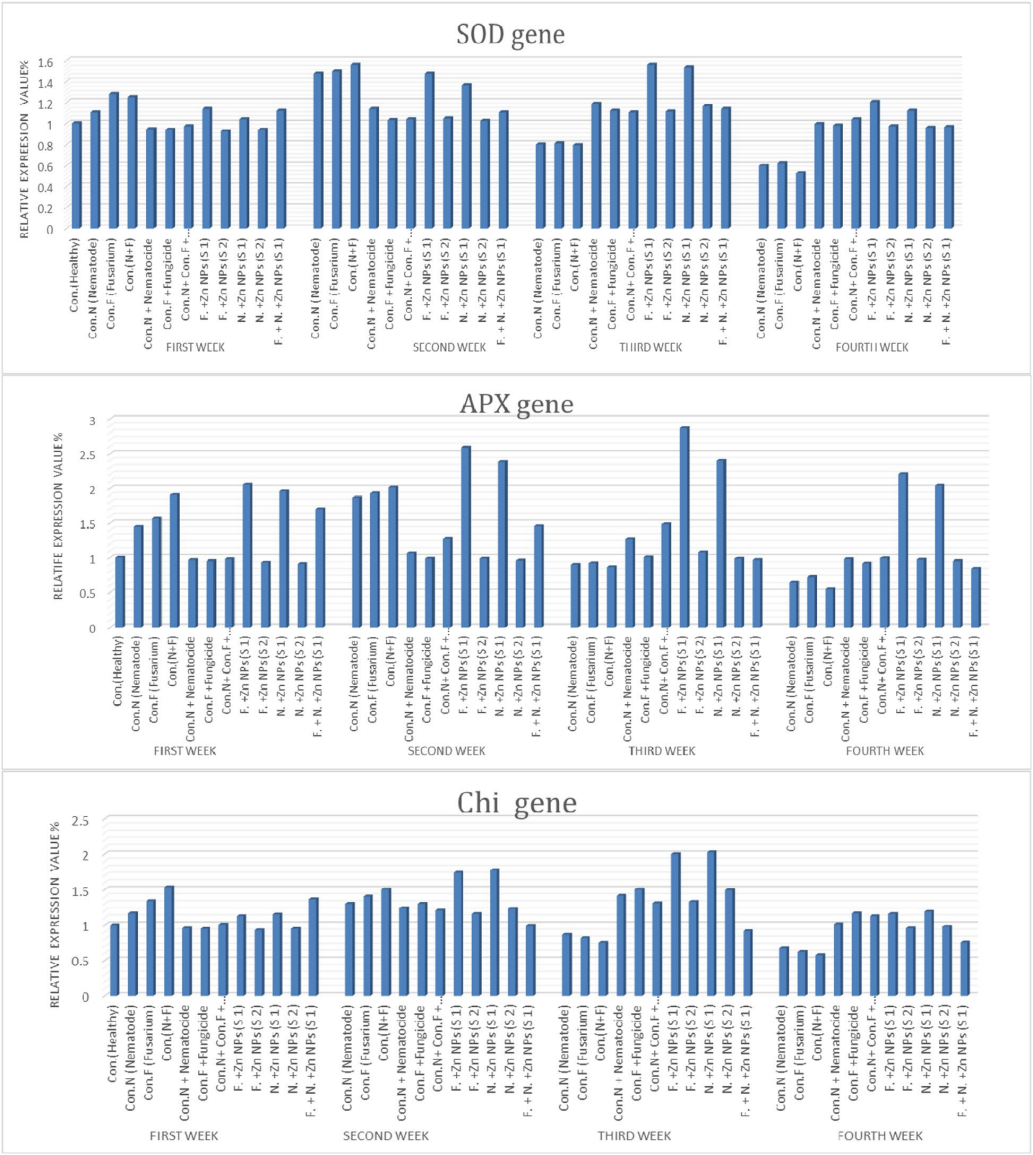
Quantitative Real-Time Polymerase Chain Reaction (qRT-PCR) validation of Chi, SOD, and APX relative genes expression in leave of pepper plants inoculated with *F. oxysporum* and *M. incognita* and treated with ZnO-NPs compare with healthy control, Uniform fungicide, and Nemacur nematicide after first, second, third and four weeks from treated

The real-time PCR comparative technique was utilized in the quantification analysis, which mathematically transforms the Cycle Threshold (CT) into the relative expression level of genes. The relative expression level of the three defense genes, Chitinase (Chi), superoxide dismutase (SOD) and Ascorbate Peroxidase (APX) were quantified by real-time PCR in the leaf of Pepper plants. The expression profile of these genes was analyzed for 4 weeks after inoculation with the pathogen (Figure 11). The results showed that the high expression level of the Chi, SOD and APX genes were noticed in the infected plants treated by ZnO-NPs (S1) after 3 weeks compared with the healthy control. These results clarified that over-expression of the three genes are directly dependent on pathogen stimulation after inoculation with* F. oxysporum* and *M. incognita*. Current results indicate that ZnO-NPs with S1 concentration can induce systemic acquired resistance.

In our study, control plants (untreated) showed no significant increase in the three genes activity whereas an increase of the expression level of them was found at different treatments during all times-points after inoculation. These data suggest that fundamental role of ZnO-NPs on activation, up-regulation of defense-related genes, and induction of defense against the infection of pathogens.

## Discussion

Plants that have been infected with *F. oxysporum* showed symptoms of infection such as wilting, loss of leaves, damping off and reddish-brown discoloration in the water-conducting tissue of the stem and roots. These symptoms are similar to what Lomas-Cano et al. mentioned about *Fusarium* infection [40]. Plants infected by *M. incognita* showed nutritional deficiency like leaves yellowing, wilting and growth supersession. These symptoms are similar with [41]. It develops to gall or swelling in roots of infected plant. Infected roots showed shorter length compared with healthy roots, plant parasitic nematodes cause lesions in plants and produce secondary infection by facilitating other organisms such as viruses, bacteria and fungi [42].

This work aimed to use the *Trichoderma longibrachiatum* to synthesis bio-zinc oxide nanoparticles (ZnO-NPs) and use it as bio-nematicide and bio-fungicide against soil-borne disease (*Meloidogyne incognita* and* Fusarium oxysporum*) infecting pepper plants. Moreover, the efficacy of ZnO-NPs on expression-defending gene change of pepper was studied using different specific primers, Chi, SOD, and APX. The current results include the morphological characteristics of *Trichoderma longibrachiatum* and *Fusarium oxysporum*, besides Amplification and sequencing of rRNA genes resulted in 516 and 504 bp long nucleotide sequence, which have been introduced in NCBI GenBank (Accession Numbers: ON203115 and ON323541p), for *T. longibrachiatum & F. oxysporum,* respectively. The observed dark green color indicated the formation of zinc oxide nanoparticles. Such a color transformation is mainly referring to changes in the metals oxidation state. As mentioned by [43] this change in color is due to reduce Zn^+2^ reduced to Zn^0^. Furthermore, the ZnO-NPs formation was confirmed by visual assessment, the absorbance has occurred at 360 nm wavelength and has great slope. This result for UV-visible spectroscopic analysis was reported by [44]. According to [45], the range of various metals-NPs in size from 2 to 100 nm in size. SEM was used to observe morphology and structure of ZnO-NPs, the results showing that ZnO NPs are spherical in nature and are agglomerates of nano crystallites, recorded at magnification of 10µm, and these results were agreeing with [46]. The TEM and EDX spectrum were used for the morphological identification of ZnO-NPs, the results showed the presence of clear signals related to zinc in (1& 9) Kev and signal related to oxygen before (1) Kev, the same result for EDX analysis was reported by [45, 46]. The FT-IR spectrum was utilized to analyze the potential biomolecules responsible for reducing zinc ions in *T. longibrachiatum* mycosynthesis nanoparticles Zn-ONPs. The findings revealed the presence of distinct and prominent peaks (3273, 2922, 2855, 1628, 1641, 1448, 1076, 1453, 1404, 1230, 1150, 883,845 and 511 cm−1), which aligned with the results reported by [23, 47, 48].

In our study, pepper infected with *M*. *incognita* and* F. oxysporum* showed significantly different physiological responses when treated *M*. *incognita* with ZnO-NPs (S, S/2) in a laboratory experiment (bioassay), the results proved that ZnO-NPs can be strong eco-nematicide and eco-Fungicide against root-knot nematode (*M. incognita)* and* F. oxysporum*, respectively. Biosynthesized ZnO-NPs can produce toxic effects in the* M. incognita and F. oxysporum*. Using ZnO-NPs gave high toxicity for *M. incognita* through 72h. ZnO-NPs proved that it has more inhibitory effect on *F. oxysporum* growth than commercial fungicide and *T. longibrachiatum*. According to [39], ZnO-NPs show great inhibitory effect on microbial activity because of their unique properties like large surface area [31]. Used SEM analysis to examine *F. oxysporum* structural changes after ZnONPs treatment and found that, after treatment with 12mg L^−1^ ZnONPs, hypha lost their smoothness and appeared swollen and crumbled and incase of untreated sample (control) observed that the hypha with typical net structure and smooth surface. This result means that ZnO-NPs Inhibitory effect on *F. oxysporum* growth may be due to deformation in the structure of fungal hypha.

The results of the present investigation indicate that there was an enhancing effect of ZnO-NPs on vegetative growth of pepper plant. It resulted in higher germination percentage (when treated with 12 µg L^−1^ ZnO-NPs), enhanced, plant height, fresh weight, dry weight and larger leaf area. The positive effect of ZnO-NPs was also significantly higher than the impact produced by the treatment with Uniform, commercial Fungicide and Nemacur (commercial nematicide). Various studies have introduced data to prove the positive impact of ZnONPs on plant growth like *Capsicum chinense* [49], and cotton [50]. The increase in vegetative growth in pepper as well as other plants reported is due to its role in maintaining the structural stability of cell membranes [51]. Zinc is involved in controlling enzymes, protein synthesis, regulating membrane function, and increasing plants’ tolerance to environmental stresses like diseases [52] according to [53] adding ZnO-NPs to the soil, has an effective role in protecting the plant against Fusarium wilt disease. ZnONPs myco-synthesized can be used as a promising and safe alternative antifungal agent against *F. oxysporum* in vitro and in vivo [53]. ZnONPs have antifungal activity against soil-borne diseases like *R. solani* , *Fusarium* sp. and *M. phaseolina* which were confirmed *in vitro* and under greenhouse conditions [54]. Nano-fertilizers have an important role in enhancing plant production besides their ability to inhibitory effects on soil-injurious microorganisms such as the root knot nematode, *M. incognita* infection [55]. ZnONPs were shown to be effective as an antagonist for various plant pathogens, such as bacteria, fungi and nematodes [56]. Leaf area can be a good indicator of plant health [57]. Nanoparticles are considered to be more effective due to their extremely small size and larger surface area, so they are more efficient as compared to their macro counterparts [58].

The results of the present study indicate that ZnONPs increase chlorophyll and total protein content. A similar study observed that, ZnO NPs increase chlorophyll and total protein content [59]. ZnONPs have increased protein content in tomato [60]. According to [61]. ZnONPs have a positive effect on protein content of callus of *Nicotiana tabacum*. [62], found that ZnO nanoparticles cause a great effect on expression of some genes encoding certain proteins, altering their expression levels either up or down. ZnO nanoparticles drastically increased the contents of chlorophyll [63]. Using of ZnO nanoparticles led to an increase contents of chlorophyll [64]. The effectiveness of the nanoparticles against the nematodes and fungi under study was the same when we repeated the laboratory experiment three months later and during greenhouse experiments, with negligible changes. This could be because the fungal biomolecules surrounding the nanoparticles provide static stability [64].

In plants, chitinases mainly play a role in the defense against pathogen attack [65]. The results of recent studies indicate that, chitinases are not only involved in defense-related processes or general stress response but also in growth and development processes. When plants are exposed to stressful biotic or abiotic environmental conditions, the production of reactive oxygen species (ROS) increases and may cause great damage to the cells. There are several antioxidant enzymes can detoxify ROS, in plants such as superoxide dismutase (SOD) which catalyses the removal of O2−by dismutating it into O2 and H_2_O_2_ and Ascorbate peroxidase (APX) enzymes which play a key role catalyzing the conversion of H_2_O_2_ into H_2_O which is known as Ascorbate-glutathione cycle [66, 67]. In our study, control plants (Untreated) showed no significant increase in the three genes activity whereas an increase of the expression level of them was found at different treatments during all times-points after inoculation. These data suggest that fundamental role of ZnONPs on activation, up-regulation of defense-related genes, and induction of defense against the infection of pathogens. The general notice is that the green synthesized nanoparticles (ZnONPs) induced the plant defense genes which rustled in high-rate production of the enzymes controlled by these genes. The high productions of these enzymes are considered as one of the acquired resistances for the plant against the plant pathogen. Our study aimed to biosynthesize ZnONps to improve sweet pepper (*Capsicum annuum* L*.*) resistance against *F. oxysporum* and *M. incognita* under greenhouse conditions and their impacts on vegetative growth parameters and physiological features.

## Conclusion

In this study, our results show that *T. longibrachiatum* possesses vital active bio-compounds, which are accountable for combating root-knot nematode, *M. incognita*, and root rot fungus, *F. oxysporum*. An optical study was done using UV-vis spectroscopy. The FT-IR results detected that phytochemicals present in *T. longibrachiatum* are responsible for the capping of ZnO NPs. The spherical morphology of the ZnO NPs was confirmed with SEM imaging and the hexagonal crystal system was confirmed with XRD. The nematicidal and fungicidal activities were determined using a direct exposure assay *in vitro*, and a high percentage of fungal inhibition and nematodes J2s mortality was observed at the higher concentration. In addition, we confirmed that by greenhouse experiments. The ZnO NPs were found to be more effective against *F. oxysprum* and *M. incognita* infected sweet pepper as compared to commercial nematicides and fungicides. Based on their incredible potency, ZnO NPs prepared from *T. longibrachiatum* could play a vital role in the field of pesticides. They could be adapted to be used in the management of invasive soil-borne diseases especially, *Meloidogyne* sp. and *Fusarium* sp.

## Data Availability

The datasets generated and/or analyzed during the current study are available in the Corresponding author repository, the isolated fungi's DNA sequences can be found in GenBank under the accession codes ON203115 and ON323541p. The corresponding author can provide all the datasets used in the analysis for the current study upon reasonable request.
